# Ectomesenchymal chondromyxoid tumor: a comprehensive updated review of the literature and case report

**DOI:** 10.1038/s41368-017-0003-9

**Published:** 2018-02-28

**Authors:** Astrid Truschnegg, Stephan Acham, Lumnije Kqiku, Norbert Jakse, Alfred Beham

**Affiliations:** 1 Department of Dental Medicine and Oral Health, Division of Oral Surgery and Orthodontics, Medical University Graz, Billrothgasse 4, 8010 Graz, Austria; 2 Department of Dental Medicine and Oral Health, Division of Preventive an Operative Dentistry, Endodontics, Periodontology, Prosthodontics, Restorative Dentistry and Implantology, Medical University Graz, Billrothgasse 4, 8010 Graz, Austria; 3 IMAH, Institute of Morphological Analytics and Human Genetics, Grabenstraße 23, 8010 Graz, Austria

## Abstract

Prompted by a unique case of an ectomesenchymal chondromyxoid tumor (ECT) of the palate in a 54-year-old female, we reviewed the English and German literature on this entity until the end of 2016 using PubMed. The search produced 74 lingual cases with a nearly equal sex distribution and a mean age of 39.3 years, and two extra-lingual cases sharing histological and immunohistological features including nodular growth, round, fusiform or spindle-shaped cellular architecture, and chondromyxoid stroma. Immunophenotyping showed the majority of cases to be positive for glial fibrillary acidic protein (GFAP), S-100 protein, glycoprotein CD57, pancytokeratin (AE1/AE3), and smooth muscle actin (SMA); in isolated cases there was molecular-genetic rearrangement or gain of Ewing sarcoma breakpoint region 1 (EWSR1) but no rearrangement of pleomorphic adenoma gene 1 (PLAG1). At present, ectomesenchymal cells that migrate from the neural crest are considered to play a pivotal role in tumor origin. All cases had a benign course, although there were three recurrences. Because of the rarity of this tumor and the need for differential diagnostic differentiation from myoepithelioma and pleomorphic adenoma, both oral surgeons and pathologists should be aware of this entity.

## Tumors: Taking stock of a rare oral growth

With the aim of deepening clinical understanding, researchers have conducted a bilingual literature review of a rare oral tumor. Due to its scarcity, relatively few instances of ectomesenchymal chondromyxoid tumor (ECT) exist in English and German medical literature, yet differentiation from other oral tumors remains important. This, combined with the presentation of a new patient with an especially rare form of the disease, led Astrid Truschnegg and her team of scientists from Austria’s Medical University Graz and IMAH to compile and assess the research conducted on ECT. The team searched papers published up to late-2016 and identified proteins commonly expressed by ECTs, patterns of cellular and tissue structure, genetic similarities, as well as a shared embryonic origin of tumor-associated cells. This review offers clinicians and researchers an accessible brief of current knowledge on a rare, yet significant oral disease.

## Introduction

Ectomesenchymal chondromyxoid tumor (ECT) is a very rare lesion almost exclusively occurring in the tongue. At present 74 lingual cases have been reported in the English and German literatures, nearly equally affecting males and females with a mean age of 39.3 years.^1–31^ The term “ectomesenchymal chondromyxoid tumor,” given in the first relevant publication, is descriptive, based on the presumption of tumor origin from migrated ectomesenchymal cells of the neural crest, and on histological and immunohistological features.^1^

Interestingly, unequivocal extralingual ECTs have been reported hitherto only twice, on the hard palate of a 13-year-old boy^32^ and in the left tonsillar bed of a 71-year-old woman.^33^ In addition, we report for the first time a palatal case in a 54-year-old woman, which extends the knowledge on the epidemiology of extralingual ECTs.

To update and check all reports on ECTs in detail and to compare lingual and extralingual cases, we carried out an exhaustive review of the relevant literature.

The literature was reviewed using PubMed for publications related to ECT in English and German languages. The following search strings were applied: ectomesenchymal chondromyxoid tumor, ectomesenchymal chondromyxoid tumor and tongue, ectomesenchymal chondromyxoid tumor and hard palate, ectomesenchymal chondromyxoid like tumor, ectomesenchymal chondromyxoid like tumor and tongue, ectomesenchymal chondromyxoid like tumor and hard palate, Ektomesenchymaler chondromyxoider Tumor, Ektomesenchymaler chondromyxoider Tumor und Zunge, Ektomesenchymaler chondromyxoider Tumor und harter Gaumen. Additionally, the references of all publications were checked for reports on ECT not found by Pubmed using the above-mentioned strings. The search was limited by the end of 2016.

## Case report

**Fig. 1:**
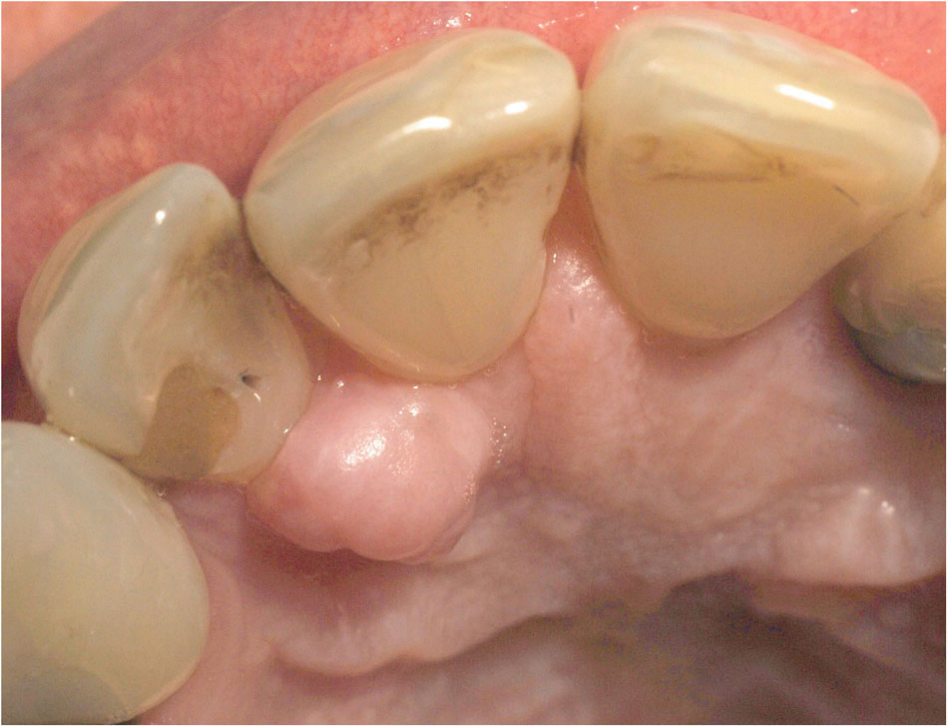
Tumor-like lesion between the first and second incisor on the palatal aspect of the right upper jaw

**Fig. 2:**
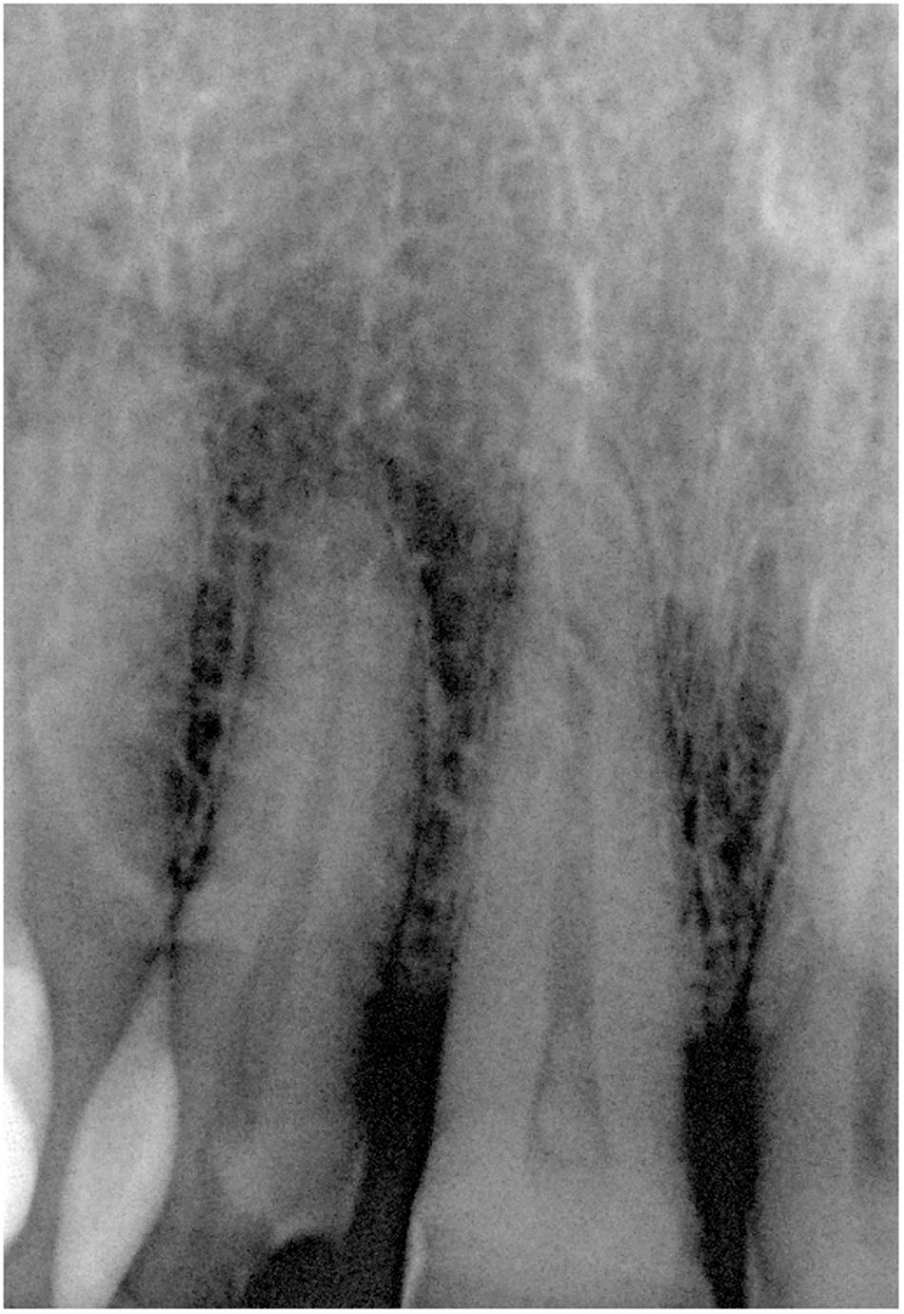
X-ray showing no involvement of the underlying bone

A 54-year-old woman presented at the Department of Oral Surgery and Orthodontics of the Medical University of Graz with a nodular lesion of the palatal gingiva. The lesion was located between the first and second incisor on the right upper jaw (Fig. 1). The patient reported that she had the lesion for a long time; it had grown larger within recent months but was painless. A pre-operative X-ray showed no tumor involvement of the neighboring maxillary bone (Fig. 2). Clinical differential diagnoses comprised epuliform lesions, most likely a fibroma or peripheral ossifying fibroma. The lesion was totally excised down to the periosteum under local anesthesia (Ultracain dental^®^ 4%, Sanofi-Aventis, Frankfurt am Main, Germany) and examined histopathologically by one of us (A.B.). A follow-up after 41 months showed no recurrence.

### Pathological examination

**Fig. 3:**
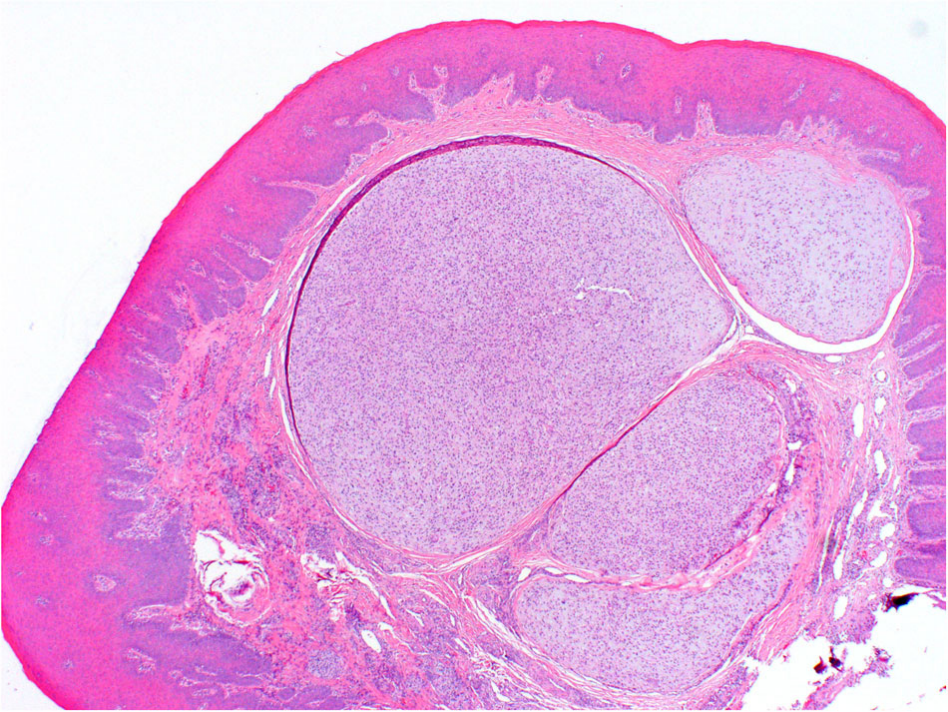
Scanning microscopy demonstrating a multinodular lesion in the stroma

**Fig. 4:**
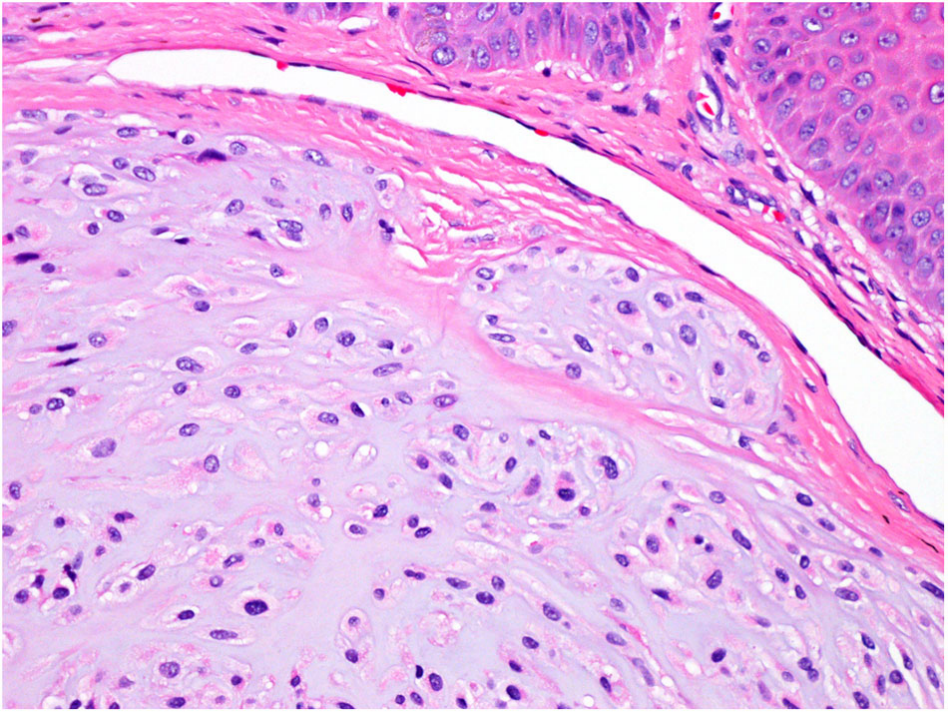
Individual tumor nodule with round to spindle-shaped cells set in myxoid stroma

**Fig. 5:**
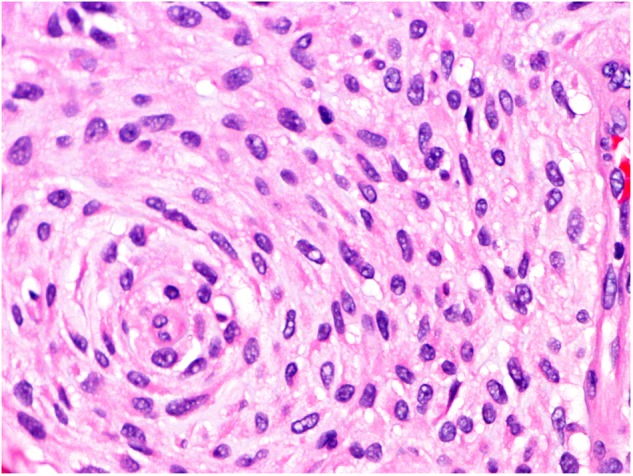
Tumor cells mainly arranged in a swirling pattern

**Fig. 6:**
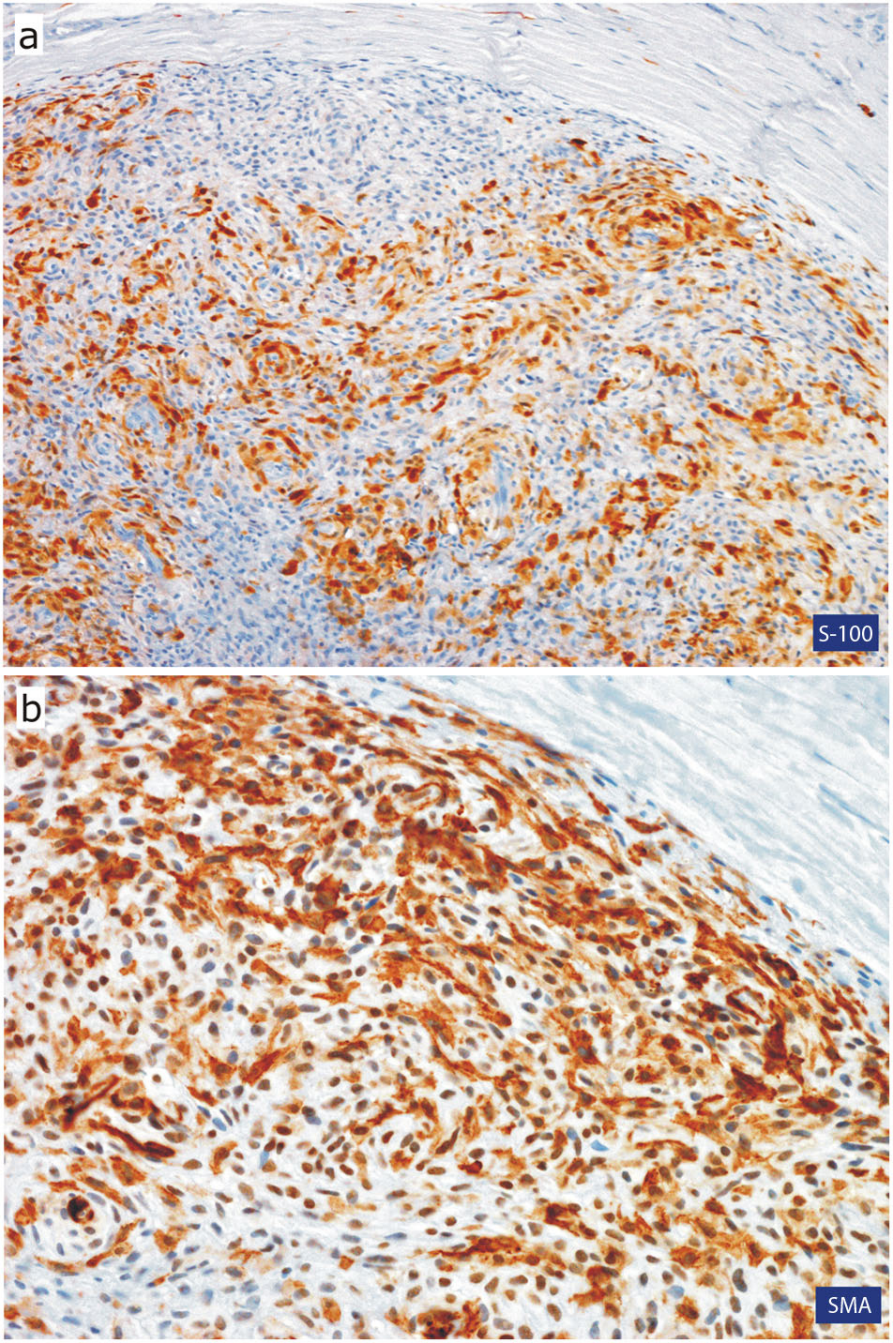
Immunohistochemical image of the tumor cells. **a** Variable expression of S-100 protein by the tumor cells. **b** Most of the tumor cells express smooth muscle actin (SMA)

The operative specimen measured 7:5:3 mm and was covered by an otherwise inconspicuous mucous membrane; the cut surface showed gelatinous tissue. Microscopically, the specimen was covered superficially by reactive hyperplastic squamous epithelium. A multinodular lesion was found in the underlying stroma (Fig. 3). The nodules varied in size and consisted of myxoid/chondroid stroma, in which many cells, mostly spindle-shaped, were embedded (Figs. 4 and 5). In general, the nodules were rich in cells, often showing eosinophilic cytoplasm. The nuclei were enlarged and hyperchromatic in some places, exceptionally with nucleoli. Perinuclear cytoplasmic vacuolization was seen in many cells. With the exception of tiny nodules, each nodule was surrounded by dense, capsule-like tissue. There were no ductal structures. Immunohistochemically the lesional cells showed variable expression of S-100 protein and smooth muscle actin (SMA). The Kiel 67 protein (Ki67)-associated cellular proliferation rate was <5%. Interestingly, there were few nodules without any S-100 protein and SMA-positive cells (Fig. 6a and b). No cells were immunoreactive for pancytokeratin, glial fibrillary acidic protein (GFAP), and cytokeratin (CK14).

## Review of the literature

### Lingual cases

**Fig. 7:**
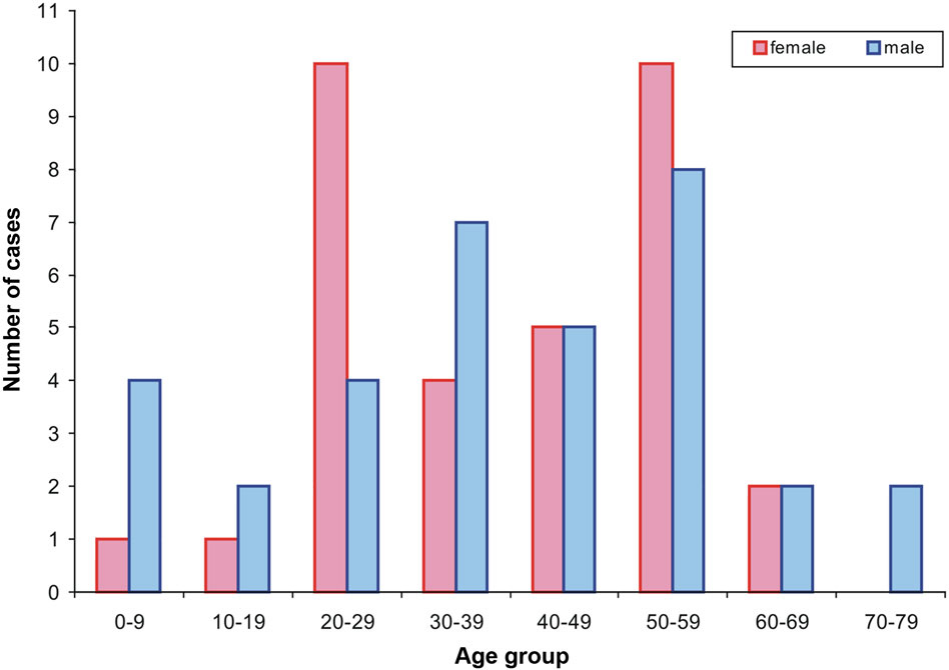
Age and sex distribution of the lingual ectomesenchymal chondromyxoid tumors

Thirty-one publications were identified in the English and German literatures dealing with 36 female and 38 male patients with an age range of 7–78 years (Table 1, Fig. 7).

**Table 1:** Chronological list of lingual and extralingual ectomesenchymal chondromyxoid tumors published in the English and German literatures

Published cases of ECT in the English and German literatures					
I. Lingual cases					
	Year	Author	Sex	Age	Localization
1	1995	Smith BC et al.	Female	23	a
2	1995	Smith BC et al.	Female	24	a
3	1995	Smith BC et al.	Female	25	a
4	1995	Smith BC et al.	Female	27	a
5	1995	Smith BC et al.	Female	36	a
6	1995	Smith BC et al.	Female	47	a
7	1995	Smith BC et al.	Female	47	a
8	1995	Smith BC et al.	Female	49	a
9	1995	Smith BC et al.	Female	57	a
10	1995	Smith BC et al.	Male	9	a
11	1995	Smith BC et al.	Male	24	a
12	1995	Smith BC et al.	Male	30	a
13	1995	Smith BC et al.	Male	31	a
14	1995	Smith BC et al.	Male	32	a
15	1995	Smith BC et al.	Male	32	a
16	1995	Smith BC et al.	Male	48	a
17	1995	Smith BC et al.	Male	53	a
18	1995	Smith BC et al.	Male	60	a
19	1995	Smith BC et al.	Male	78	a
20	1996	Kannan R et al.	Female	51	a
21	1996	Kannan R et al.	Male	21	d
22	1996	Kannan R et al.	Male	33	a
23	1996	Van der Wal JE & Van der Waal I	Female	25	a
24	1999	Carlos R et al.	Male	16	p
25	2003	de Visscher JGAM et al.	Female	42	a
26	2003	de Visscher JGAM et al.	Male	39	a
27	2003	Ide F et al.	Female	52	a
28	2004	Kaplan I et al.	Female	57	d
29	2004	Kaplan I et al.	Male	26	a
30	2005	Woo VLK et al.*	Female	22	d
31	2006	Goveas N et al.	Female	57	a
32	2008	Seckin D et al.	Female	56	a
33	2009	Pires FR et al.	Male	9	a
34	2009	Pires FR et al.	Male	16	a
35	2009	Pires FR et al.	Male	33	a
36	2009	Portnof JE et al.	Male	41	a
37	2010	Angiero F	Female	27	a
38	2010	Chopra R et al.	Female	47	a
39	2010	Nikitakis NG et al.*	Male	45	a
40	2010	Seo SH et al.	Male	8	p
41	2010	Seo SH et al.	Male	65	a
42	2011	Leeky M et al.	Male	7	a
43	2011	Muenst S et al.	Male	52	a
44	2011	Sengul D et al.	Female	28	a
45	2012	Guzman JMP et al.	Female	35	a
46	2012	Guzman JMP et al.	Female	68	a
47	2012	Pak MG et al.	Female	16	a
48	2012	Tsai SY et al.	Male	41	a
49	2013	Closman JJ et al.	Male	23	a
50	2013	Yoshioka Y et al. 2013	Female	66	a
51	2014	Cardin MJ et al.	Male	43	p
52	2014	Kale H et al.	Female	7	a
53	2015	Aldojain A et al.	Male:female = 4:3	Mean age = 45.8 Range: 7–57	a
54	2015	Aldojain A et al.			a
55	2015	Aldojain A et al.			a
56	2015	Aldojain A et al.			a
57	2015	Aldojain A et al.			a
58	2015	Aldojain A et al.			a
59	2015	Aldojain A et al.			p
60	2015	Tajima S and Koda K	Female	24	a
61	2016	Argyris PP et al.	Female	22	n.l.a.
62	2016	Argyris PP et al.	Female	31	n.l.a.
63	2016	Argyris PP et al.	Female	33	n.l.a.
64	2016	Argyris PP et al.	Female	53	n.l.a.
65	2016	Argyris PP et al.	Female	54	n.l.a.
66	2016	Argyris PP et al.	Female	54	n.l.a.
67	2016	Argyris PP et al.	Female	57	n.l.a.
68	2016	Argyris PP et al.	Male	53	n.l.a.
69	2016	Argyris PP et al.	Male	54	n.l.a.
70	2016	Argyris PP et al.	Male	59	n.l.a.
71	2016	Argyris PP et al.	Male	70	n.l.a.
72	2016	Laco J et al.	Male	58	a
73	2016	Laco J et al.	Male	56	a
74	2016	Schep LA et al.	Male	51	a

II. Extralingual cases					
	Year	Author	Sex	Age	Localisation
1	2012	Gouvêa AF et al.	Male	13	Hard palate anterior right
2	2016	present case	Female	54	Hard palate region 11/12
3	2016	Stecco et al.	Female	71	Left tonsillar bed

*a*, anterior; *d*, dorsal; *p*, posterior; *n.*l.*a.*, no location available;

* published as myoepithelioma

#### Histogenesis

In their seminal paper on ECT, Smith et al.^1^ favored the histogenesis of these tumors from ectomesenchymal cells that had migrated from the neural crest. They backed up this theory with embryonic considerations and immunohistochemical examinations demonstrating GFAP in 73% of the cases tested with a monoclonal antibody, and in 100% with a polyclonal antibody. Yoshioka et al.^24^ were able to confirm the origin of ECT in ectomesenchymal cells derived from neural crest by demonstrating the expression of homeobox protein-transcription factor (Nanog), GFAP and microtubule associated protein 2 (MAP2) in cell cultures and positivity for octamer binding protein 3/4 (OCT3/4), transcription factor Sox2, Nanog, MAP2 and CD 105mRNAs in real-time polymerase chain reaction (RT-PCR) analysis. Moreover, with immunohistochemical methods, Laco et al.^30^ showed expression of transcription factor Sox10 in one of their two cases, so supporting the proposed neural crest theory.

#### Nature

ECT is currently classified as an entity by the World Health Organization (WHO),^34^ but has morphological and immunohistochemical properties in common with myoepithelioma, and to a lesser extent with pleomorphic adenoma.

Looking for classical light microscopic features pointing to pleomorphic adenomas, Ide et al.,^6^ Chopra et al.,^14^ and Closmann et al.^23^ were unable to find ductal structures in ECTs. In addition, Argyris et al.^29^ could not find molecular genetic rearrangement of pleomorphic adenoma gene 1 (PLAG1) in seven cases.

However, stimulated by previous genetic examinations on soft tissue myoepithelial tumors^35^ Argyris et al.^29^ successfully demonstrated rearrangement or gain of Ewing sarcoma breakpoint region 1 (EWSR1) in 3 of 11 and 8 of 9 ECTs, respectively, whereas Laco et al.^30^ failed to find an EWSR1 rearrangement in two such tumors.

#### Clinical features

##### Age and sex

The seven-case series of Aldojain et al.^27^ does not provide data on age and sex of the individual patients, so that detailed relevant data were only available for the remaining 67 cases (90.5%) of the tumors of the tongue. The age range was between 7 and 78 years with a mean age of 39.3 years. For male patients, the age range was 7–78, for a mean age of 38.7 years, while the age range of female patients was 7–68 years, for a mean age of 40 years. The majority of the male cases (*n* = 20, 52.6%) appeared in the fourth, fifth, and sixth decades, which is in some contrast to female cases, which most often occurred in the third and sixth decades (in total 20 cases, 55.5%). Interestingly, no female was older than 68, though the oldest male was 10 years older. The 67 cases involved 34 males and 33 females, for a nearly equal sex distribution of 1.03:1.

##### Location

In 63 cases (85.1%) data concerning the location were given.^1–28,30,31^ Fifty-six lesions occurred on the anterior part of the tongue, and four lesions on the posterior part; the remaining three lesions were only reported to be on the dorsum of the tongue but without further positional information. Of the “anterior cases” 26 could be found in males and 24 in females. The remaining six cases were listed by location only without gender.^27^ Three of the four “posterior cases” occurred in males. The fourth case was given only by location but without gender.^27^

##### Clinical aspect

In 47 cases the size of the lesions could be determined by clinical inspection; they ranged in size from 3 to 50 mm (mean 13.6 mm). Of them, 45 were located on the anterior aspect of the tongue and ranged in size from 3 to 50 mm (mean 13.3 mm)^1–3,5–7,9–24,26,28,30,31^; the two lesions on the posterior tongue each had a diameter of 20 mm.^4,16^ In the 31 cases (exclusively anterior lesions) that described the impression of palpation, 19 cases were described as firm^1,2,5,7,9,10,12–14, 16,17,20,23,26,31^, 1 as soft^1^, 1 cystic,^1^ 1 firm-cystic^1^, 4 elastic,^3,18,19,22^ 2 non-tender^24,28^, 1 tender^20^, 1 firm-cystic^1^, 1 soft-cystic.^6^

##### Clinical differential diagnoses

The reported differential diagnoses included various mainly mesenchymal lesions^36^, whose spectrum expanded when histopathology was taken into consideration.^27^ However, judging the macroscopic illustrations in all the publications on the basis of our own clinical experience, we suggest fibroma, neurofibroma, myoepithelioma, and pleomorphic adenoma as the most likely clinical diagnoses.

#### Preoperative procedures

##### Imaging

Imaging studies of the tumors were available for four cases. Sonograms of two cases revealed one hyperechoic and one hypovascular lesion.^17,18^ Computed tomography (CT) scan without contrast in a further case showed a partially cystic mass^25^ and magnetic resonance imaging (MRI) in the fourth case featured low-level signals and contrast enhancement.^24^

##### Fine needle aspiration biopsy (FNAB)

In six cases a preoperative FNAB was performed, which, however, was always inconclusive with the final diagnosis.^5,14,17,18,25,26^

#### Therapy

In 54 of 74 cases the lesions were removed in toto by excision^1–3,5–22,24–26,28,30,31^, whereas in the remaining cases there was no information on the nature of the surgical procedure.^2,4,13,27^

#### Pathology

##### Histopathology

For 74 lesions, only partial information was available for the following parameters: 61 lesions were described as circumscribed^1–3,5–8,10,11,13–23,26,28–31^ and 37 as lobular/nodular^1,2,7,8,12–14,17,19,22–24,26,28,30^; 44 lesions revealed a growth pattern forming cords, strands, and net-like structures.^1,3,5,6,8,14,16,17,20–27,31^ All lesions showed a mixture of varying numbers of round, spindled, ovoid, and fusiform cells.^1–3,5–31^ Among them four lesions also exhibited epithelioid cells.^29^ Every single lesion was characterized by a chondromyxoid stroma.^1–3,5–31^ Entrapment of the adjacent skeletal muscle was found in 44 lesions.^1,2,5,8,12–19,21,26–29^

##### Immunohistopathology

A great many antibodies were applied in the examination of ECTs, among which neurogenic markers, cytokeratins and myogenic markers play an outstanding role. Most ECTs were immunoreactive for GFAP (85.7%), S-100 protein (80.4%), and CD57 (77.4%).^1,2,6,7,15,18,20,27,30^

Of the cases tested with the pan-cytokeratin antibody AE1/AE3, 55.3% stained positive^1,8,9,11,13,20,27,28,31^, while cytokeratin antibodies directed against a single subclass of cytokeratins almost always were negative. Using an antibody to SMA, 51.1% of 47 cases exhibited positive immunostaining.^1,11,12,15,17,20,23,24,27,28,30^, which was exceeded by the muscle-specific actin marker HHF-35, which stained 3 of 3 cases^2^ An immunoreaction for desmin could be detected in 30% of the cases evaluated.^2,5,11,15,25,27,31^ Since transformation-related protein 63 (p63) is thought to be one of several myoepithelial markers, it is remarkable that 31.8% of the cases examined were at least immunoreactive for this marker.^8,12,15,24,27^

##### Electron microscopy

Ultrastructural studies were performed in only three exclusively anterior cases. The lesions of a 27-year-old female and a 58-year-old male exhibited tumor cells with lobulated/concave nuclei, homogeneous chromatin distribution and one to two small nucleoli, dilated endoplasmic reticulum, intermediate filaments, and a partial basal lamina; desmosomes or condensed thin filaments could not, however, be demonstrated.^1,30^ Tumor cells in the case of a 51-year-old woman probably were poorly sampled and so showed no intracytoplasmic matrix production, but pinocytotic vesicles, a well-developed rough endoplasmic reticulum and tight junctions.^2^

##### Differential diagnosis

Histopathologically, myoepithelioma^1,2,8,14,25,29^, and pleomorphic adenoma^1,8,14,29^ were the most relevant differential diagnosis because of their pronounced histological similarities.

#### Follow-up

In 33 (44.6%) of 74 lingual cases unequivocal data were available for follow-up. The follow-up period was between 2 and 240 months.^1,6^ Three of the 33 cases (9.1%) revealed recurrences appearing after 3 months, 19 months^1^, 60 months.^12^ Metastatic deposits were never reported.

### Extralingual cases

At present only three extralingual cases including the present one and concerning two female patients and one male patient aged between 13 and 71 years have been reported. Detailed data are given in Table 1.

In all three cases tumor cells were immunoreactive for S-100 protein, in two cases for GFAP^32,33^, and in only one case for cytokeratin^33^ and SMA (present case).

A further case reported in the hard palate was not acceptable due to lack of immunohistochemical data.^37^

## Discussion

The authors of the first publication on the tumors under discussion suggested the descriptive name “ectomesenchymal chondromyxoid tumor” (ECT) based on morphological and immunohistochemical parameters and, more importantly, on histogenetic and embryologic considerations.^1^ In 2005, ECT was adopted by the World Health Organization as a diagnostic term, which is still in use.^34^

The location of ECTs in the tongue may be explained by their embryological development, in which migrating ectomesenchymal cells of the neural crest of branchial arches play an important role.^24^ In this context, the nearly exclusively lingual occurrence of ECTs can be elucidated by the development of the anterior 2/3 of the tongue from the first branchial arch.^38^ The same considerations are applicable to the cases involving the hard palate. By contrast, the posterior third of the tongue, where ECTs have been found very rarely, originates from the second, third, and fourth branchial arches.^38^ This is in accordance with a recently published ECT of the left tonsillar bed/parapharyngeal space^33^, which are known to originate from the second branchial arch.^38^

Although the number of extralingual ECTs is extremely small^32,33^, the clinical data mirror those of the lingual cases; i.e. the former also occurred in both sexes (one male and two female patients), with an age distribution of 13–71 years and a mean age of 46 years.

In the two palatal cases, x-rays showed no pathology in the underlying bone.^32^ Since the lingual cases were set in purely soft tissue, these locations did not lend themselves to radiographic studies. Probably because of its location, only the peritonsillar/parapharyngeal lesion was investigated by CT scan, showing a central calcification and no enhancement of contrast medium.^33^ This is paralleled by an MRI analysis of a lesion of the tongue, also without contrast medium enhancement.^24^

As in the overwhelming majority of the cases in the tongue, two of the three extralingual cases were initially excised completely. In the third such case, by contrast, the lesion was first biopsied and then excised^32^, to establish a firm diagnosis prior to surgery, identically to the therapeutic procedure by Closmann et al.^23^ in a lingual case. The extralingual tumors showed no recurrences within a short period of 6 months’ follow-up^32,33^, and a long period of 41 months (present case). This is in contrast to the tumors of the tongue, which recurred in three cases^1,12^, which may be due to incomplete initial excision.

The extralingual specimens ranged in size from 7 to 30.5 mm (mean 20.7 mm), and so were larger than the lingual ones (3–50 mm; mean 13.9 mm).

Whether localized in the hard palate or in the tonsillar bed/parapharyngeal space, the lesions were histopathologically characterized by circumscribed unencapsulated proliferation with lobular architecture and by monomorphic rounded, spindled, stellate, or polygonal cells set in a chondromyxoid stroma. Since their features are identical to tongue lesions, it can be said that the morphological pattern of ECT is independent of tumor localization.

Like the lingual lesions, the extralingual ones have to be discriminated clinically from a range of different lesions frequently occuring in the oral cavity^32,33,36^, whereas pathohistologically mainly pleomorphic adenomas and myoepitheliomas have to be considered in the differential diagnosis. In general, in classical histopathology pleomorphic adenomas are characterized by ductal structures, whereas these features have never been reported for ECT or should not appear in proper myoepitheliomas.^35,39^ On the other hand, chondroid changes of the stroma may be seen in pleomorphic adenomas and ECTs, which is in contrast to typical myoepitheliomas.^39^

Immunohistochemically, all three extralingual cases expressed S-100 protein, which could also be detected in more than three-quarters of the lingual cases. The same can be said with regard to the reactivity for GFAP in two cases.^32,33^ The negativity for GFAP in our case may be explained by the use of a monoclonal antibody, which is a finding also seen in lingual cases^8,16,17,27^ and in contrast to examinations with polyclonal antibodies. The impossibility of demonstrating pancytokeratins in the two palatal cases^32^ mirrors the variability of pancytokeratin expression in tongue lesions.

Both palatal cases differ in the immunoreaction for SMA. This discrepancy may be explained by the use of different antibodies to SMA, the source of which is not given in the SMA-negative case by Gouvêa et al.^32^ or by the tumors themselves because only 51.1% of lingual cases are also SMA positive. Since pleomorphic adenomas and myoepitheliomas are known to express the antigens mentioned above^35,39^, immunohistochemistry alone does not allow a strict differentiation of these lesions from ECT.

Despite close histological and immunohistological similarities, for several reasons it is unlikely that ECT is a subtype of pleomorphic adenoma. ECTs are mainly located in the anterior part of the tongue, an area that is devoid of salivary glands, which are considered to be the origin of pleomorphic adenomas. Moreover, ductal structures have never been found in ECT.^6,14,23^ In any case, genetic examinations showed no rearrangement of PLAG1 in 7 cases.^29^

Very recent genetic studies, however, have demonstrated rearrangement or gain of EWSR1^29^, implying that ECT is genetically somehow similar to myoepithelioma.

Summarizing, based on histopathological, immunohistpathological, and common genetic features, some degree of relationship between ECT, myoepithelioma, and pleomorphic adenoma may be supposed.

In the setting discussed above in detail, our unique extralingual case in the hard palate of a woman differs substantially in its location from the vast majority of ECTs reported, but is similar in terms of histopathology, immunohistopathology, and biological behavior.

### Addendum

After finishing our study by the end of 2016 in 2017 the further relevant paper on ectomesenchymal chondromyxoid tumors was published: “Kato MG, Erkul E, Brewer KS, Harruff EE, Nguyen SA, Day TA. Clinical features of ectomesenchymal chondromyxoid tumors. A systematic review of the literature*. Oral Oncol* 2017;67:192–197”.
